# An Alkaline Based Method for Generating Crystalline, Strong, and Shape Memory Polyvinyl Alcohol Biomaterials

**DOI:** 10.1002/advs.201902740

**Published:** 2020-09-24

**Authors:** Mohammad Ali Darabi, Ali Khosrozadeh, Ying Wang, Nureddin Ashammakhi, Halima Alem, Ahmet Erdem, Qiang Chang, Kaige Xu, Yuqing Liu, Gaoxing Luo, Ali Khademhosseini, Malcolm Xing

**Affiliations:** ^1^ Center for Minimally Invasive Therapeutics (C‐MIT) University of California Los Angeles CA 90095 USA; ^2^ Department of Bioengineering University of California Los Angeles CA 90095 USA; ^3^ Department of Radiological Sciences David Geffen School of Medicine University of California Los Angeles CA 90095 USA; ^4^ Department of Mechanical Engineering University of Manitoba Winnipeg R3T 5V6 Canada; ^5^ Terasaki Institute for Biomedical Innovation Los Angeles CA 90024 USA; ^6^ Department of Physical & Environmental Sciences University of Toronto Scarborough Toronto Ontario M1C 1A4 Canada; ^7^ Institute of Burn Research State Key Lab of Trauma Burns and Combined Injury Southwest Hospital Third Military Medical University Chongqing 400038 China; ^8^ Université de Lorraine CNRS Institut Jean Lamour (UMR 7198) Campus Artem 2 allée André Guinier‐BP 50840 Nancy Cedex F54011 France; ^9^ Department of Chemistry Kocaeli University Umuttepe Campus Kocaeli 41380 Turkey; ^10^ Department of Biomedical Engineering Kocaeli University Umuttepe Campus Kocaeli 41380 Turkey; ^11^ Department of Chemical Engineering University of California Los Angeles CA USA

**Keywords:** biomaterials, catheters, hydrogels, injectable electronics, microfluidics, polyvinyl alcohol, shape memory

## Abstract

Strong, stretchable, and durable biomaterials with shape memory properties can be useful in different biomedical devices, tissue engineering, and soft robotics. However, it is challenging to combine these features. Semi‐crystalline polyvinyl alcohol (PVA) has been used to make hydrogels by conventional methods such as freeze–thaw and chemical crosslinking, but it is formidable to produce strong materials with adjustable properties. Herein, a method to induce crystallinity and produce physically crosslinked PVA hydrogels via applying high‐concentration sodium hydroxide into dense PVA polymer is introduced. Such a strategy enables the production of physically crosslinked PVA biomaterial with high mechanical properties, low water content, resistance to injury, and shape memory properties. It is also found that the developed PVA hydrogel can recover 90% of plastic deformation due to extension upon supplying water, providing a strong contraction force sufficiently to lift objects 1100 times more than their weight. Cytocompatibility, antifouling property, hemocompatibility, and biocompatibility are also demonstrated in vitro and in vivo. The fabrication methods of PVA‐based catheters, injectable electronics, and microfluidic devices are demonstrated. This gelation approach enables both layer‐by‐layer and 3D printing fabrications.

## Graphical abstract

This work introduces a facile strategy to induce crystallinity and produce physically crosslinked polyvinyl alcohol biomaterials with high mechanical properties, stable chemistry, resistance to injury, and shape memory properties. Fabrication methods for producing hydrogel‐based catheters, injectable electronics and microfluidic devices are discussed, using 3D printing and layer‐by‐layer methods.

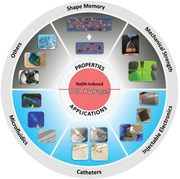

## 1 Introduction

Engineered biomaterials have been increasingly recognized as a promising solution to overcome major hurdles in healthcare.^[^ ^1^ ^]^ Development of stretchable and strong biomaterials with long‐term performance, chemical stability, and multifunctional properties can facilitate translating them into clinical and industrial applications.^[^ ^2^ ^]^ Meanwhile, biomaterials which are adaptable to 3D printing and microfluidic technologies are highly beneficial to provide controlled synthesis, versatile properties, and accurate biomimicking solutions.^[^ ^3^ ^]^ Hence, new methods to form biomaterials are necessary to make progress in advancing the field.

Hydrogels can be made of a diverse group of synthetic and natural materials and can be categorized into physical and chemical gels.^[^ ^4^ ^]^ The formation of crystallites is among the strongest physical crosslinking methods in forming hydrogels,^[^ ^5^, ^6^ ^]^ which combined with hydrogen bonding can provide strength and elasticity. Polyvinyl alcohol (PVA) with tunable crystallinity has been largely used to improve mechanical properties of biomaterials used for tissue engineering^[^ ^7^ ^]^ and biomedical applications such for wound dressings,^[^ ^8^ ^]^ contact lenses,^[^ ^9^ ^]^ vascular grafts,^[^ ^10^ ^]^ artificial meniscus,^[^ ^11^ ^]^ and vitreous substitute.^[^ ^12^ ^]^

PVA hydrogels are commonly synthesized through two mechanisms comprising chemical bonding using a cross‐linker such as glutaraldehyde and/or physical crosslinking such as cyclic freeze–thaw methods.^[^ ^13^ ^]^ PVA hydrogels with high mechanical properties and chemical stability require low water to PVA ratio^[^ ^2b^ ^]^ as well as dense PVA networks.^[^ ^2a^ ^]^ The strength and toughness of conventional PVA hydrogels with high swelling ratios are greatly diminished when they absorb water.^[^ ^2b^ ^]^ Accordingly, having dense polymer networks and higher degree of crystallinity are the major contributing factors in forming strong PVA hydrogels.

In this paper, we aim to develop a method to form a strong and stretchable PVA biomaterial with crystalline and dense networks. We hypothesize that applying alkaline hydroxide in a high concentration into a highly dense PVA polymer network can rapidly induce crystallinity. According to our proposed mechanism, first, the deprotonation of hydroxyl groups of PVA is achieved via the basic attack of OH^−^ ions. Subsequently, the complexation can be formed between O^−^ group and the free Na^+^, facilitating the mobility of PVA chains to be stretched and aligned and form crystalline domains. This chain organization will be stabilized and crystalline domains will be developed by replacing the complex with hydrogen bonds as soon as they are in contact with water. Moreover, the higher amount of hydrogen bonding and crystallite formation lead to the expelling of water molecules, leading to a low swelling ratio without sacrificing the elasticity. To prove our hypothesis and demonstrate potential applications, we characterized the proposed process and comprehensively studied the thermal, physical, mechanical, chemical, and biological properties of our proposed physically crosslinked PVA hydrogel (PVA‐H). Finally, benefiting from the current approach, a water‐induced shape memory biomaterial, and artificial muscle system with the PVA‐H are designed. To reveal potential applications of our novel PVA biomaterial in biomedical applications, biocompatibility of PVA membranes are evaluated both in vitro and in vivo and their applications in fabricating stretchable and robust injectable electronics, catheters, and microfluidic devices are elucidated.

## 2 Results and Discussion

### 2.1 Preparation and Mechanism

**Figure 1:**
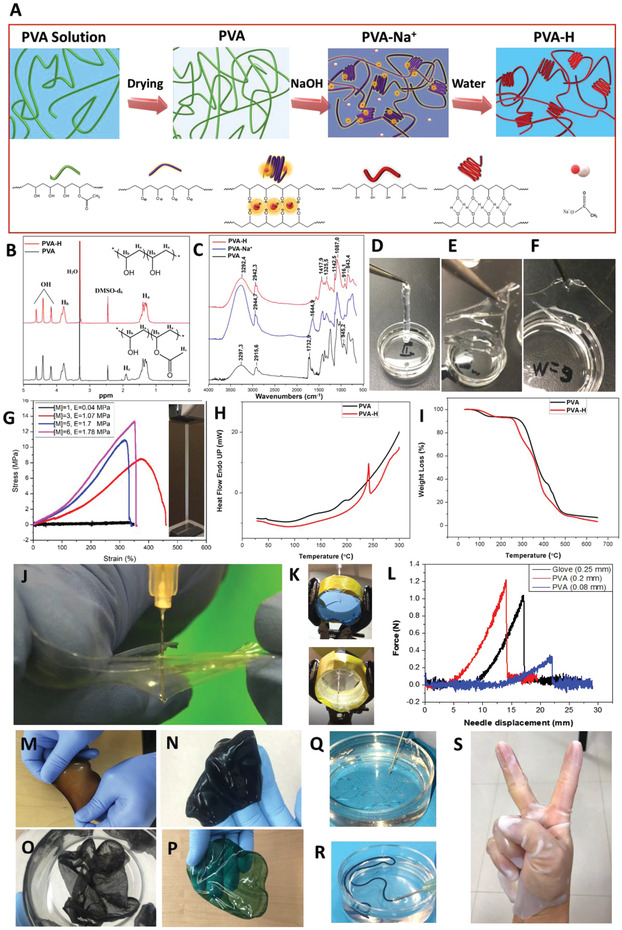
A) Schematic illustration of PVA‐H preparation and proposed mechanism (PVA‐Na^+^: PVA immersed in a NaOH solution; PVA‐H: crosslinked PVA hydrogel). B) ^1^H‐NMR spectrum of PVA and PVA‐H in DMSO‐d_6_ solvent. C) FTIR spectra of PVA, PVA‐Na^+^ (intermediate step) and PVA‐H. D–F) Photos of PVA‐Hs made with different molarities of NaOH solution: D) 1 m forming gel with the thickness of 0.05 mm, E) 3 m forming gel with the thickness of 0.1 mm, and F) 6 m forming gel with the thickness of 0.1 mm. G) Stress–strain curves and elastic moduli of PVA‐Hs made with different NaOH concentrations. The inset shows a PVA strip during tensile test. H) DSC curves of a dried PVA membrane and a PVA‐H. I) TGA curves of PVA and PVA‐H. J) Demonstration of the resistance of PVA‐H to needle puncture (Video S1, Supporting Information). K) Optical images of a medical glove and a PVA‐H membrane tighten in a customized holder for measuring the resistance against needle puncture in Figure 1L. L) The force–displacement graph when an injection needle was pushed onto the medical glove with 0.025 mm and PVA‐H samples with 0.08 mm and 0.2 mm thicknesses. M–P) PVA‐Hs incorporating different nanomaterials including M) MNPs, N) graphene, O) CNT, and P) PVA‐H coated with PANI. Q) PVA solution injection in a NaOH bath to make fibers. R) PVA/CNT injection in NaOH bath to make PVA/CNT fibers. S) PVA solution can be coated on a mold and crosslinked to make different shapes such as a glove.

A 100 mg mL^−1^ solution of PVA (*M*_w_ = 205 000 g mol^−1^, Aladdin) was prepared by dissolving PVA powder in deionized water at 90 °C under magnetic stirring overnight. A specified amount of the PVA solution was poured or coated on the surface of a Petri dish or mold and was allowed to dry completely. The PVA‐H was then formed by immersing the dried PVA film in a high‐concentration solution of strong alkaline hydroxide (NaOH) for 10–40 min (depending on the thickness of films), followed by water treatment. To explain the proposed mechanism, we called NaOH‐treatment as the intermediate step, where the polymer chains are organized (**Figure** **1**A). Afterward, the insoluble membrane was immersed in deionized water to remove ions and to stabilize crosslinked crystalline PVA networks (crystallites). All PVA biomaterials were prepared using 6 m solution of NaOH unless otherwise stated. The thickness of membranes can be readily tuned by changing the volume of the initial PVA solution.

In the intermediate step, we aimed to organize the stacked polymer chains to form crystallization and crosslinking with our new approach by using a high‐concentration solution of NaOH. The NaOH attack leads to the hydrolysis of the ester group of the acetate moieties from the polymer backbone as confirmed by nuclear magnetic resonance (NMR) spectroscopy, where the ^1^H signal of the acetate methyl group proton signals named H_c_ disappeared (Figure 1B).^[^ ^14^ ^]^ We suppose that applying a strong alkaline hydroxide into a dried PVA film results in two sequential events in the intermediate step. First, OH^−^ of the alkaline hydroxide attacks the hydroxyl groups of PVA, resulting in disrupted hydrogen bonds and deprotonation of the hydroxyl groups of the PVA chain. Afterward, the newly formed O^−^ groups in PVA interact with free Na^+^ ions to form complex. This new complexation allows the polymer chain to move freely via new network as shown in Figure 1A. This new organization in the intermediate step, results from the conformation of polymer chains that form parallel and stretched macromolecules. These two sequential processes facilitate the PVA crystallization as the intermediate step induces the preorganization of the polymer chain as depicted in Figure 1A. Followed by immersing NaOH‐treated PVA (PVA‐Na^+^) in water, crystalline domains are stabilized, and PVA‐H are formed. In this final step, adding water will remove Na^+^ ions, and O^−^ will be protonated and crystalline domain will be permanently stabilized, leading to a strong PVA‐H (Figure 1A). In following we characterized each step with several experimental methods.

The interaction of the hydroxyl groups in PVA with OH^−^ moieties of the highly concentrated NaOH solution leads to the acid‐base reaction [Equation (1)], and it is confirmed by the difference between the pKa values which is 1.5 (14 for H_2_O/OH^−^ and 15.5 for R—OH/R—O^−^; we chose to use the value generally used for CH_3_—CH_2_—OH/CH_3_—CH_2_—O^−^, as no data is available regarding the pKa of PVA). This confirms that two thirds of OH groups of the PVA backbone are deprotonated and they can be further complexed by Na^+^ ions.^[^ ^15^ ^]^

(1) $$\text{R}\;-\;\text{OH}\;+\;{\text{Na}}^{+}\;{\text{OH}}^{-}⇄\;\text{R}\;-\;{\text{O}}^{-}\;{\text{Na}}^{+}+\;{\text{H}}_{2}\text{O}$$

Fourier transformed infrared (FTIR) spectra are provided in Figure 1C which depicts the spectra of the PVA (pristine film), PVA‐Na^+^ (intermediate step), and PVA‐H (crosslinked PVA). After the hydrolysis, the fingerprint peak related to acetate groups between 1750–1735 cm^−1^ disappeared which confirmed the full efficiency of the acid‐base reaction. Indeed, this peak is linked to the stretching C=O from acetate group which is in agreement with the NMR data (Figure 1B).

The appearance and sharpness of the peak at about 1142 cm^−1^ for the PVA‐H and PVA‐Na^+^ confirmed the re‐organization of the polymer chains after the reaction with NaOH (Equation 1). Indeed, the intensity of this peak is directly related to the crystalline portion of the polymeric chains.^[^ ^16^ ^]^ The increase in the degree of crystallinity is then enlightened by the increase of the intensity of this peak, which is the fingerprint of the crystallinity for the PVA.^[^ ^16a^ ^]^ Moreover, the intensity of the peak attributed to the CH_2_ wagging and twisting at around 1018 cm^−1^ decreased after the hydrolysis. This suggests that the C—H bonds of the CH_2_ groups of the backbone are less free to deform out of the plane, which indicates that the polymer chains are more confined, preventing the free deformation of the C—H bonds mainly owing to the van der Waals interactions.

### 2.2 Mechanical Properties

The effects of the NaOH concentration on the mechanical and gelation properties of PVA‐H are shown in Figure 1D–G. The crosslinking of PVA film with 3–6 m solution of NaOH leads to successful gelation (Figure 1D,E). However, 1 m solution of NaOH resulted in the formation of a very weak and thin membrane (Figure 1D) with a very low elastic modulus (0.04 MPa), indicating unsuccessful crosslinking, since the concentration is too low to stoichiometrically deprotonate OH groups of the PVA and only strong basic media, pH >12, can deprotonate PVA to form a strong metal‐binding ligand.^[^ ^17^ ^]^ According to the stress‐strain curves, all samples exhibited elastic behavior when they were stretched up to 100% strain. In addition, all samples demonstrated excellent ductility, exceeding 350% of their original length. Further comprehensive tensile experiments were carried out to probe the effect of dehydration on the mechanical properties of PVA‐Hs (Figure S1, Supporting Information). The results show that the continuous dehydration of a PVA‐H leads to denser PVA chains, and thus gradual increase of its elastic modulus and mechanical strength.

To evaluate the effectiveness of alkaline metal hydroxide agents, tensile properties of PVA‐Hs (0.1 mm thickness) crosslinked with NaOH, KOH, and LiOH, were studied (Figure S1A, Supporting Information). The use of LiOH led to an unstable and weak hydrogel, showing an ineffective crosslinking. On the other hand, NaOH‐ and KOH‐crosslinked membranes exhibited high mechanical strengths with ultimate strengths above 13 MPa and elastic moduli above 1.7 MPa. This is because LiOH is a weak base due to its low dissociation constant in water. LiOH bond is described as having maximum covalent character because of Fajans’ Rule, which finally hampers an efficient reactivity of the OH^−^.^[^ ^18^ ^]^

### 2.3 Crystallinity

The increase in crystallinity was further confirmed by differential scanning calorimetry (DSC) to calculate the PVA crystalline fraction. Typical DSC for PVA and PVA‐H films are shown in Figure 1H. A considerable increase in crystallinity from 7.35% to 56.9% was observed, confirming the efficiency of our new crosslinking approach. This effect contributes to the enhancement of the mechanical properties of the PVA‐H compared to the PVA. The change in the crystallinity degree was also confirmed by X‐ray diffraction measurement shown in Figure S2, Supporting Information. Thermogravimetric analysis (TGA) was performed to evaluate PVA and PVA‐H thermal properties (Figure 1I). The first region (around 100 °C) is related to the evaporation of water molecules entrapped in the polymer. The evaporation of water molecules is delayed for the PVA‐H sample as seen in the change of the slope due to the crystalline structure, which decreases the diffusion of H_2_O through the materials.^[^ ^19^ ^]^ The second region corresponds to the hydrogen bonding break and the degradation of the PVA backbone. PVA shows higher stability in this region which is due to the lower amount of the crystallites. Crystallites are known to degrade earlier than entangled amorphous chains as the crystalline organization with the polymer enables first the chain separation and second their degradation. This is impossible for amorphous polymers as the entanglement of the chains hinder their free mobility and lead to degradation of the material, which require higher amounts of energy.^[^ ^20^ ^]^ In the formal freeze–thaw method, the phase separation and crystallization are caused by water molecules during the repeated freezing and thawing.^[^ ^21^ ^]^

### 2.4 Other Properties

The scanning electron microscope (SEM) of a PVA‐H film is presented in Figure S3, Supporting Information, showing very fine PVA networks. Water content of PVA‐H is 33 ± 3% and the equilibrium swelling ratio of PVA‐H is 0.9 ± 0.1. Such a low water content and swelling ratio along with the transparency of this PVA‐H offer a unique opportunity for its application in designing biomedical devices such as contact lenses. The long‐term stability of PVA‐H was evaluated by storing PVA‐H strips in water for 2 years without observing any tangible changes in their physical and mechanical properties, which qualitatively demonstrates their long‐term stability.

Provided by NaOH‐induced gelation method, PVA‐Hs performed excellent resistance to damage by pointed and sharp objects (Video S1, Supporting Information). For demonstration, a 21G needle was pushed onto PVA membranes and a nitrile glove (VWR International, LLC) to measure the puncture forces. The PVA membrane showed higher resistance to puncture by needle compared to nitrile glove (Figure 1J–L).

Furthermore, current gelation method allows the incorporation of different nanomaterials, such as magnetic nanoparticles (MNPs), graphene, and carbon nanotubes (CNTs), into PVA‐Hs that enables us to produce functional hybrid biomaterials with adjustable thicknesses (Figure 1M–P). PVA‐H membranes can also be coated with conducting polymers such as polyaniline (PANI) using solution‐processing methods for flexible and stretchable energy storage or sensor applications.^[^ ^22^ ^]^ For instance, the color of a PANI‐coated PVA‐H film immediately changed from green (emeraldine salt form of PANI) to blue/purple (emeraldine base form of PANI) when it was transferred from an acidic (1 m H_2_SO_4_) to a basic solution (1 m NaOH), and it recovered its original color upon returning it to H_2_SO_4_ solution, which can be employed in the development of optical pH sensors (Figure S5 and Video S2, Supporting Information). The decoration of PVA‐H with PANI showed negligible effect on its mechanical strength. As a demonstration of the strength of PVA‐H/PANI film, a dried PVA‐H/PANI strip (≈0.5 g) was used to lift 9 kg weight, 18 000 times more than its own weight (Video S2, Supporting Information).

Different molds can be used before the crosslinking step to form complex shapes such as a glove (Figure 1S). The mechanical strength and water content of PVA‐Hs can also be tuned by controlling the dehydration level of PVA before the crosslinking step. For instance, PVA‐H fibers were immediately formed by injecting a PVA solution (200 mg mL^−1^) into the NaOH bath (Figure 1Q). Due to the less dense network of PVA solution, these PVA fibers were mechanically weaker than PVA‐Hs that were dried before the gelation. Figure 1R illustrates the formation of PVA/CNT fibers with improved elastic modulus by injecting PVA/CNT solution (≈100 mg mL^−1^ PVA and 10 mg mL^−1^ CNTs) into the NaOH solution, indicating the possibility of direct solution‐to‐hydrogel preparation of PVA/CNT.

Our results show that our PVA‐H introduces improved mechanical properties and low swelling ratio compared to conventional PVA hydrogels in the literature (Table S1, Supporting Information).^[^ ^2^, ^23^ ^]^ Additional merits of current strategy include the ability to incorporate variety of nanomaterials and the possibility to fabricate various shapes comprising strong and stretchable films and tubes with an adjustable thickness as small as several micrometers. It should be noted that this method can be applied for PVA polymers with molecular weights higher than 31 000, since it provides longer chains and higher chain entanglements (Figure S6, Supporting Information).

### 2.5 Shape Memory Effect and Artificial Muscle

The as‐prepared PVA‐H films can recover more than 90% of large plastic elongation upon immersing in water. For instance, a PVA‐H strip with a length of 26 mm (width 8 mm, thickness 0.1 mm) was stretched to 100 mm after tensile tests. Upon immersing the strip in water for 40 min, the length was decreased to 32.6 mm, which is 91% recovery in plastic deformation, indicating its shape memory characteristic.

**Figure 2:**
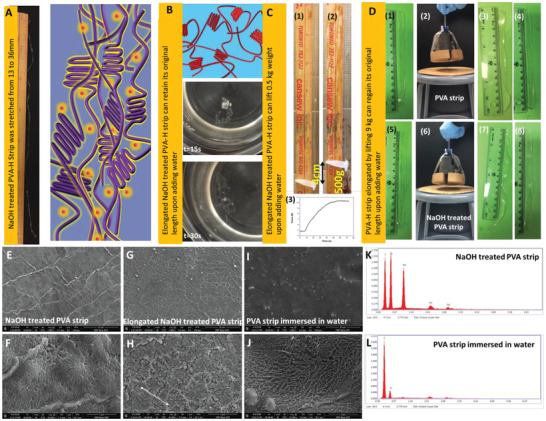
Demonstration of the shape memory property of the PVA‐H and its potential to develop artificial muscles. A) The PVA‐H strip was immersed in NaOH solution and elongated from 13 mm to 36 mm. Schematic shows the disruption of hydrogen bonds by the basic attack in the PVA‐H strip, enhancing the mobility of the PVA chains. B) Then, it was immersed in water, which regained its original length in a few seconds (Video S3, Supporting Information). C) An elongated PVA strip which was immersed in NaOH solution can provide a strong contraction force upon adding water which can lift a 0.5 kg weight up to 4 cm (1,2) Contraction force measured during the addition of water to the same PVA‐H strip (used in C1 and C2) using tensile machine (Video S4, Supporting Information) (3). D) The PVA‐H strip (1) can carry 9 kg (2), leading to the permanent elongation (3), which was regained upon immersing in water (4). A PVA‐H strip treated with NaOH solution (5) which was lifting 9 kg (6), underwent a large elongation (7) which can be regained upon adding water (8) (Video S5, Supporting Information). E,F) SEM images of PVA‐H immersed in NaOH solution. G,H) SEM images of a NaOH‐treated PVA‐H film, which was completely stretched into plastic region (Arrow shows stretching direction). I,J) SEM images of a PVA‐H film, which recovered to its original length after adding water. K,L) EDX spectra of samples presented in (E) and (I) respectively.

PVA‐Hs can recover from plastic deformation by immersion in water. In addition, they can demonstrate a shape memory behavior when immersed in NaOH solution. The PVA‐H becomes soft and its ductility will be significantly increased might be because PVA becomes deprotonated and hydrogen bonds are destroyed (**Figure** **2**A). As such, the NaOH‐treated PVA‐H strip can be stretched to the plastic region with less force than a non‐treated one because of its lower stiffness. This is attributed to disruption of hydrogen bonds by the basic attack, which leads to an enhanced chain mobility in the PVA‐H.

Supplying water to the PVA‐H will remove Na^+^ ions and O^−^ will be re‐protonated, leading to the return of previously disrupted hydrogen bonds. This process results in the recovery of the plastic elongation, representing the proposed mechanism of the shape memory (Figure 2B and Video S3, Supporting Information). For example, a NaOH‐treated PVA‐H strip with an initial length of 13 cm was stretched to 36 cm (176.9% strain; Figure 2A). Upon immersing the strip in water for 40 s, its length returned to 14 cm, revealing 95.7% recovery of its large plastic deformation or shape memory behavior (Figure 2B and Video S3‐part 1, Supporting Information). In a similar manner, another NaOH‐treated PVA strip with an initial length of 13 cm was stretched to 27 cm (107.7% strain). The length of the strip decreased to 13 cm after immersing it in water for 70 s, indicating full recovery of its plastic deformation (Video S3‐part 2, Supporting Information).

The quick recovery of large strain in an elongated strip by supplying water provides a strong contraction force which can be utilized in artificial muscle applications.^[^ ^24^ ^]^ Water‐assisted recovery of previously disrupted hydrogen bonds in the NaOH‐treated PVA‐H strip provides energy to roll back the plastic elongation. So, when a weight is attached to an elongated strip, this recovered energy provides contraction force to lift a weight. For instance, a NaOH‐treated PVA‐H strip (width: 10 mm, thickness: 0.3 mm and length: 13 cm) was manually stretched and was attached to a 0.1 kg weight reaching the final length of ≈32 cm (167% strain). The contraction force generated by supplying water to the strip was sufficient to lift the 0.1 kg weight up to 10 cm in ≈45 s with an average speed of ≈2 mm s^−1^ (Figure S7 and Video S4, Supporting Information). The same strip was used to lift the 0.5 kg weight up to 4 cm in ≈65 s (Figure 2C). It should be noted that the generated contraction force in the strip was slightly larger than the weight of attached objects (*Mg*), i.e., 0.98 N and 4.91 N for the 0.1 kg and 0.5 kg, respectively. As such, the work done on the 0.1 kg and 0.5 kg weights by the strip during their hoist were ≈98.1 mJ and ≈196.2 mJ, respectively. To further measure the peak of the contraction force in the strip, the NaOH‐treated and pre‐stretched strip was installed on the tensile test grips with a 4.91 N preloading and without further stretching. Upon supplying water to the strip, the force was gradually increased up to 10.4 N in about 50 s (Figure 2C (3)), demonstrating that the strip can lift even heavier weights. Figure 2D and Video S5, Supporting Information further illustrate the ability of PVA‐H (treated and not treated with NaOH) in carrying 9 kg weight which led to permanent elongation in PVA‐H. We also showed that these deformations can be reversed upon adding water.

SEM images of a NaOH‐treated PVA‐H are shown in Figure 2E,F. For comparison, SEM images of a NaOH‐treated PVA‐H after it was stretched to its plastic region are provided in Figure 2G,H. It can be observed that the surface morphology of the NaOH‐treated PVA‐H is more flat with cracks perpendicular to the stretching direction. According to the SEM images in Figure 2I,J, the surface morphology of the PVA‐H film after it was immersed in water is like those of freshly prepared PVA‐Hs (Figure S3, Supporting Information). This observation further supports the shape memory effect of the PVA‐H that when treated with NaOH, it can recover from large plastic elongations by adding water. The EDX spectrum of the NaOH‐treated PVA‐H reveals the embedment of NaOH ions in PVA network (Figure 2K), whereas the EDX spectrum of the PVA‐H confirm the removal of NaOH ions after immersing in water (Figure 2L).

### 2.6 Biocompatibility

To expand the potential applications of this material for biomedical applications, biocompatibility of PVA‐Hs was investigated. Two cell types, human keratinocytes (HaCaT) and fibroblasts (3T3), were cultured with medium extracted from PVA‐Hs. CCK‐8 kit cell proliferation‐toxicity assay and cell viability assay were performed to test PVA‐H's potential toxicity. Figure S8A,C, Supporting Information show live/dead staining for HaCaT and 3T3 cells on day three post‐seeding, respectively. Green fluorescence indicates living cells by cell‐permeant calcein AM and red fluorescence indicates dead cells by EthD‐1. Red fluorescence labeled cells were almost invisible for all images. According to Figure S8B,D, Supporting Information, PVA‐H films were cytocompatible after a period of 5 days and exhibited significant cell proliferation levels comparable to the control group.

Protein and bacterial anti‐fouling performance was evaluated with bovine serum albumin (BSA, life, USA) as protein model and *E. coli* as bacteria model. Figure S8E,F, Supporting Information, represent FITC‐BSA protein adsorption of the PVA‐H films and cover glasses (control). Results suggested that developed PVA‐H membranes can resist protein adsorption better than the control group. Furthermore, according to Figure S8G,H, Supporting Information, the of number adhered *E. coli* on PVA‐H membranes was significantly lower than that in the control group (*p* < 0.001).

Red blood cell‐induced in vitro hemolysis is a reliable and important indicator to evaluate the hemocompatibility. The hemolysis rate (HR) related to controls and PVA‐H membranes are summarized in Table S2, Supporting Information. HR below 5% were considered to be non‐hemolytic according to ISO 10993–41999. The HR of the PVA‐H group was far less than 5%, which suggest they do not induce hemolysis when in contact with red blood cells (Table S2, Supporting Information).

Implantation of biomaterials will normally induce a foreign body reaction.^[^ ^25^ ^]^ This may persist as a chronic inflammatory response^[^ ^26^ ^]^ to degradation products, such as seen with biodegradable materials.^[^ ^27^ ^]^ To assess the occurrence of foreign body reaction, PVA‐H membranes were subcutaneously implanted in mice. Figure S9, Supporting Information, represents hematoxylin and Eosin staining of the tissue slices of these membrane‐containing tissue explants, 4 weeks post‐operatively. With such stable PVA‐Hs, we did not observe any increased inflammatory reaction and giant cells. Only mild inflammatory reaction were observed in the form of the presence of some macrophages in the near vicinity of implants. Accordingly, no evident mature fibrous tissue capsule was formed around the PVA‐H at this time, and no tissue or cell necrosis occurred. In addition, in our 1‐month in vivo study by implantation in the subcutis of mice, no evidence of materials degradation was observed.

### 2.7 Layer‐by‐Layer Fabrication and Applications

**Figure 3:**
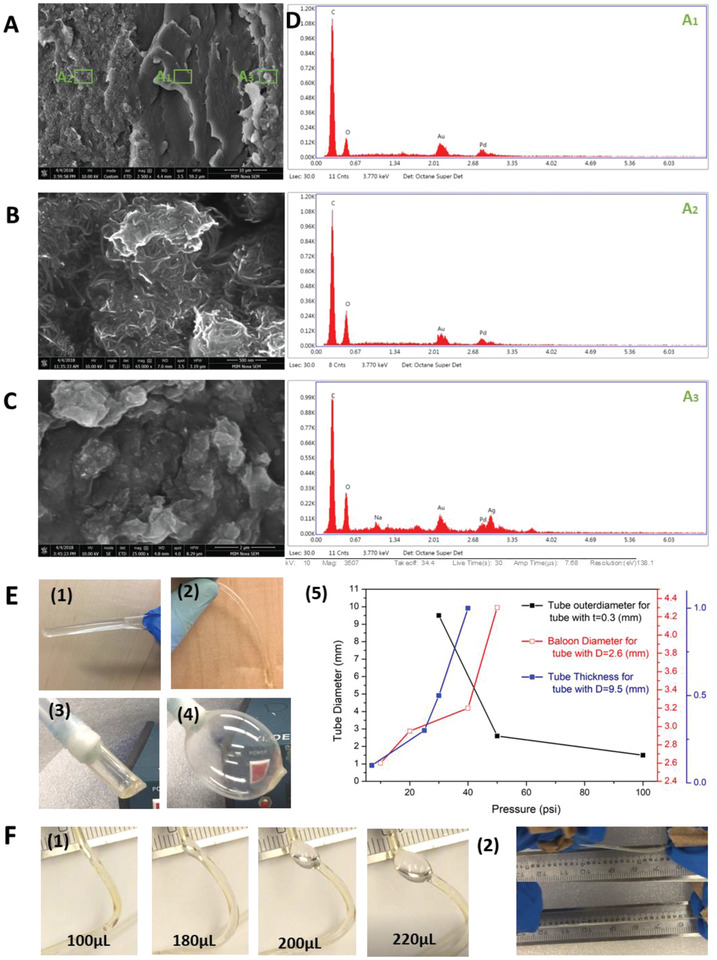
A) Cross‐sectional SEM images of a triple‐layered composite membrane of PVA‐H/CNTs, pristine PVA‐H and PVA‐H/AgNPs (left to right). High resolution SEM images of B) PVA‐H/CNTs, and C) PVA‐H/AgNPs. D) EDX spectra of three selected areas in the composite membrane (A_1_, A_2_, and A_3_). E) Images (1) and (2) show PVA‐H tubes with (9.5, 0.3) and (1.5, 0.2) (diameter, thickness [mm]), respectively. Images (3) and (4) show a PVA‐H tube (1, 9.5) connected to compressor and blown to four times larger than its original diameter before bursting. (5) The effect of tube diameter on the burst pressure for tubes made with 0.3 mm thickness (black); the effect of applied pressure on the diameter of balloon catheter (red); the effect of tube thickness on the burst pressure of tubes made with 9.5 mm diameter (blue). F) A small tube (1.5, 0.2) (1) was pumped with water using a syringe, and it was able to store up to 220 μL of water before bursting. (2) Demonstration of the elasticity of a PVA‐H tube (1.5, 0.2).

This crosslinking approach allows layer‐by‐layer assembly and incorporation of various nanomaterials with PVA into a single film with adjustable thickness. For example, a triple‐layered composite membrane of PVA/silver nanoparticles (AgNPs), pristine PVA, and PVA/CNT were successfully fabricated. SEM image of the membrane cross‐section shows the effective attachment and assembly of all layers with micro‐sized thickness (**Figure** **3** and Table S3, Supporting Information).

Given the excellent mechanical properties and facile fabrication method, developed PVA biomaterials can potentially be used to make catheters and implants. Catheters are important tools in minimally invasive delivery of therapeutics^[^ ^2f^ ^]^ promoting quick recovery time after procedures such as coronary artery bypass.^[^ ^28^ ^]^ Catheters have been developed for delivery of new shear thinning occluding material for the treatment of brain aneurysms.^[^ ^29^ ^]^ Catheters were used for delivering drugs in the brain and^[^ ^30^ ^]^ liver,^[^ ^31^ ^]^ as well as delivering pancreatic islets via the portal vein.^[^ ^32^ ^]^ The most common cause for the failure of long‐term indwelling catheters is the formation of biofilm and crystalline deposits. Colonized bacteria on the catheter surface trigger serious infections in the urinary tract which can also lead to blockage in catheters, resulting in trauma and pain.^[^ ^33^ ^]^ Meanwhile, there is extensive research on biofilm prevention with methods such as coating of conventional catheters with amphiphilic and antifouling polymers^[^ ^34^ ^]^ or using hydrogels.^[^ ^35^ ^]^ Other critical properties required in designing catheters include strong elastic properties, protein and bacterial antifouling, biocompatibility, and durability.

Tubes with tunable mechanical properties, diameters and thicknesses can be built by coating PVA solution on a metal rod followed by drying and gelation with a high‐concentration solution of NaOH (Figure 3E). To ensure a uniform coating of PVA solution, a metal rod was immersed in a PVA solution and rotated using an electric motor during the drying step, which can be repeated to attain a desired tube thickness. The diameter and thickness of PVA tubes can be adjusted such that they can be blown with fluid or gas, revealing their potential application as balloon catheters. For instance, Figure 3E (3,4) displays a PVA tube with an outer diameter of 9.5 mm and a thickness of 1 mm which was blown to four times bigger than its original diameter. Figure 3E (5) shows the input pressure required to increase the diameter of a tube with an initial diameter of 2.6 mm to a balloon with a specific size. Since the resistance against internal pressure is vital in designing catheters, we also investigated the effect of tube diameter and thickness on the burst pressure (Figure 3E (5)). Increasing the tube thickness leads to higher tolerance against internal pressure, whereas increasing the tube diameter deceases the burst pressure. For example, a stretchable tube with an outer diameter of 1.5 mm and thickness of 0.3 mm can resist internal pressure up to 689 kPa, which is also comparable with Triple‐Lumen central venous catheters used in healthcare,^[^ ^36^ ^]^ human saphenous vein^[^ ^37^ ^]^ and human artery.^[^ ^38^ ^]^ Figure 3F shows a small tube with a diameter of 1.5 mm that was pre‐filled with around ∼40 µL of water and was able to store up to an additional 220 µL of water before bursting.

Accordingly, we are able to design catheters with compliant and non‐compliant balloons by controlling the diameter and thickness of PVA tubes, using a low swelling and low water content hydrogel.

### 2.8 3D Printing Fabrication and Applications

Another application of the proposed crosslinking approach is in designing hydrogel‐based injectable electronics and implants capable of tolerating high shear forces during injection while maintaining their physical structure and function after injection. Developing injectable electronics can pave the way for controlled drug delivery, continuous monitoring, manipulation, and follow up of a therapy.^[^ ^2^, ^39^ ^]^ Mechanical strength and the ability to resist large shear forces during injection as well as seamless integration of electronic with tissues are crucial properties for injectable electronics.^[^ ^40^ ^]^ By combining layer‐by‐layer assembly and printing, it is also possible to build miniature electronics having required injectability and stretchability.

**Figure 4:**
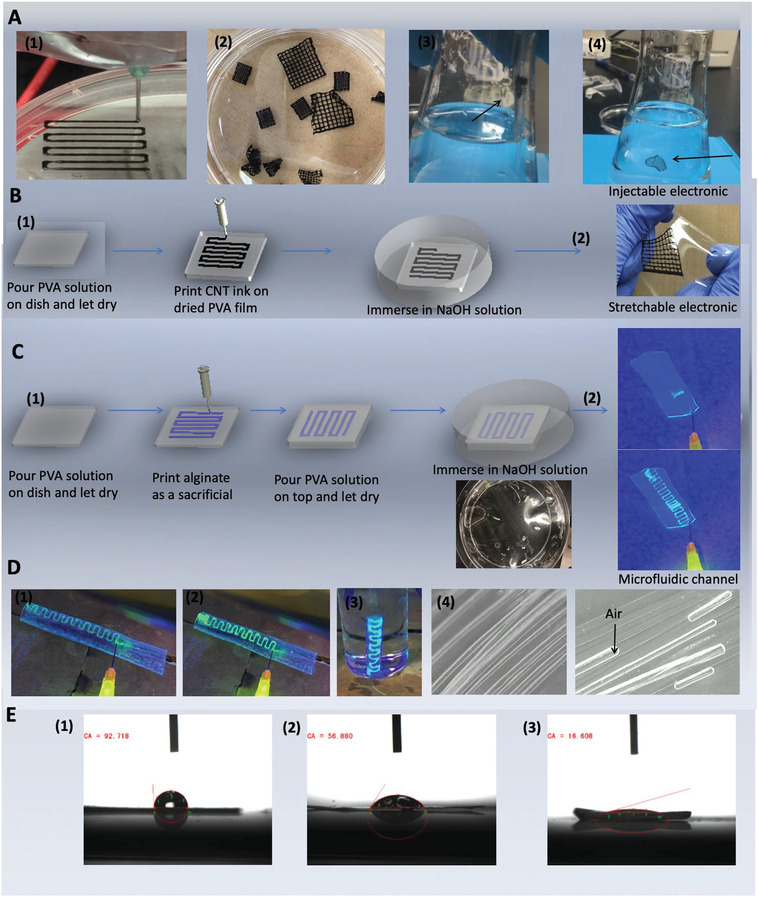
A) PVA/CNT solution while printing (1). 3D printed hydrogels after gelation (2). 3D printed hydrogel (1 cm2) was injected into water through a small pipette tip with 400 µm diameter (Video S6, Supporting Information) (3 and 4). B) Schematic of PVA/CNT conductive ink printed on a thin PVA membrane (1). Image of a stretchable electronic (2) (Video S7, Supporting Information). C) Synthesis of microfluidic channels inside a membrane wall (the inset shows the microfluidic channels built in a PVA membrane is immersed in NaOH solution) (1). Photos of a microfluidic channel under UV radiation before (top) and after (bottom) injection of water‐containing dye to the channel (2) (Video S8, Supporting Information). D) Photos of a microfluidic channel with a diameter of 300 μm that was built inside the wall of a PVA‐H tube (9.5 mm diameter). Water‐containing dye was injected to the channel (1 and 2). A microfluidic channel inside a PVA‐H tube was injected with a dye solution and was placed in water under UV radiation (3). Observation of water and trapped air inside the channels of microfluidic arrays built within a PVA‐H film under microscope (microchannels with 20 µm diameter) (4) (Video S8, Supporting Information). E) Contact angles (CA) measured for PDMS; CA = 92.7° (1). PVA (before gelation) dried sample; CA = 56.9° (2). Dried PVA‐H; CA = 16.6° (3) (contact angles were captured 5 s after the droplet touched the films).

To fabricate a conductive PVA‐H/CNT mesh, PVA/CNT ink (Experimental Section in Supporting Information) was printed on a Petri dish, followed by physical crosslinking using NaOH solution (**Figure** **4**A (1,2)). The as‐prepared mesh was readily peeled off from the printing substrate to form a strong and stretchable conductive mesh, offering numerous applications in wearable sensors and printable electronics. This mesh can preserve its integrity even after being pushed through the tiny hole of a pipette tip (Figure 4A (3,4), Video S6, Supporting Information), revealing its injectability. Furthermore, PVA can be used as a substrate for printing conductive materials to produce stretchable electronics (Figure 4B (1)). Figure 4B (2) displays a conductive mesh effectively assembled on a PVA film (Video S7, Supporting Information shows the performance of the printed mesh on a PVA film).

Other applications that can benefit from employing our strong and biocompatible materials include developing functional organ‐on‐a‐chip devices that can be used as alternative disease models and drug testing platforms^[^ ^41^ ^]^ or implantable microfluidic devices.^[^ ^42^ ^]^ Considering that only limited number of materials are being used in designing microfluidic devices, such as polydimethylsiloxane (PDMS), which is associated with the problem of absorption of hydrophobic drugs,^[^ ^43^ ^]^ and polymethylmethacrylate (PMMA), which is rigid, strong hydrogels with stretchability, low water content, and high transparency can change the trajectory of progress in this field.

PVA‐based microfluidics with adjustable channel sizes can be fabricated with the aid of layer‐by‐layer assembly and 3D printing. Here, a high‐concentration solution of alginate was employed as a sacrificial material to create microfluidic channels. According to Figure 4C, the alginate solution was printed on a dried PVA film and then was covered with another layer of PVA solution, followed by the drying step. Upon treating the composite film with NaOH solution to crosslink PVA and subsequent water treatment, the printed alginate was readily removed by gently pressing the film and passing water through the channels. To demonstrate the successful fabrication of channels inside a PVA film, a solution of fluorescein sodium salt was injected into the channels under UV light (Figure 4C (2), Video S8, Supporting Information).

Microfluidic channels can also be engineered in nonplanar PVA surfaces using this approach (Figure 4D (1–3), Video S8, Supporting Information). The optical microscope images in Figure 4D (4) show arrays of microfluidic channels (≈20 µm diameter) inside a PVA‐H film produced using human hair as a sacrificial material in a similar layer‐by‐layer procedure. To provide a clear exhibition of microchannels inside a PVA‐H film, it was placed in water so that water can be perfused into channels, during which air was also trapped inside channels. Capillary‐induced contact angle between the trapped air and water inside microchannels is the indication of their hydrophilic surface. The water contact angle (Figure 4E) for PDMS, dried PVA film before gelation, and dried PVA‐H were found to be 92.7°, 56.9°, and 16.6 °, respectively. In contrast to PDMS, the low contact angle of dried PVA‐H reconfirms its high hydrophilicity, which is a critical property for microfluidic systems. The conventional PDMS‐based microfluidic systems typically require further surface treatments to improve their hydrophilicity and cell compatibility. The high elastic modulus, high hydrophilicity, biocompatibility, and biofilm antifouling of the proposed PVA‐H could open up new opportunities for the lab‐on‐a‐chip systems, microfluidic bioprinting,^[^ ^44^ ^]^ and implantable microfluidic devices.^[^ ^42^, ^45^ ^]^

## 3 Conclusion

Here, we developed a facile crosslinking method to fabricate strong and stretchable PVA biomaterials useful for several biomedical applications. Treating dense stack of PVA polymer with a high‐concentration solution of alkaline metal hydroxide can physically crosslink the polymer and increase the crystallinity to form an elastic material with a low water content and swelling ratio. Furthermore, by using this strategy, we can incorporate different nanomaterials to prepare functional hybrid biomaterials having different shapes with the aid of 3D printing and layer‐by‐layer assembly. Developed PVA‐Hs were characterized by shape memory property with the capability to recover 90% of plastic deformation with a contraction force sufficient to lift objects 1100 times more than their own weights which can be used for actuator and artificial muscle applications. This material is cytocompatible, hemocompatible, and biocompatible with antifouling properties. Given their strong mechanical properties, low water content, chemical stability, capability of incorporating different nanomaterials, and 3D printability, this new material can be used to develop catheters, vascular grafts, articular cartilage, corneal replacement materials, and contact lenses. Beyond medical applications, the material can also potentially used for food packaging.

## Conflict of Interest

The authors declare no conflict of interest.

## Author Contributions

M.A.D. and A.Kho contributed equally to this work. M.A.D. developed the material and designed the project. M.A.D. and A.Kho. developed the idea, designed and implemented the experiments, and wrote and revised the manuscript. Y.W conducted in vitro and in vivo tests. N.A., H.A., and A.E. assisted in chemical characterizations, writing and revising the manuscript. Q.C, K.X. and Y.L. assisted in the puncture resistant experiment and prepared PVA‐H glove. G.L. and M.X. supervised in vivo tests. A.K. and M.X. supervised the project and edited the manuscript.

## Supporting information

Supporting Information

Supplemental Video 1

Supplemental Video 2

Supplemental Video 3

Supplemental Video 4

Supplemental Video 5

Supplemental Video 6

Supplemental Video 7

Supplemental Video 8
