# Supplementary material for: An Alkaline Based Method for Generating Crystalline, Strong, and Shape Memory Polyvinyl Alcohol Biomaterials
Source: Adv Sci (Weinh). 2020 Sep 24;7(21):1902740. doi: 10.1002/advs.201902740 (PMC7610272; doi:10.1002/advs.201902740)
Supplement: Supplementary file 1 — Supporting Information [file ADVS-7-1902740-s001.pdf]

© 2020 Wiley-VCH GmbH

ADVANCED SCIENCE
Open Access

Supporting Information

for *Adv. Sci.,* DOI: 10.1002/advs.201902740

A New Method for Producing Crystalline, Strong, and Shape Memory Polyvinyl Alcohol Based Biomaterial

Mohammad Ali Darabi, Ali Khosrozadeh, Ying Wang, Nureddin Ashammakhi, Halima Alem, Ahmet Erdem, Qiang Chang, Kaige Xu, Yuqing Liu, Gaoxing Luo, Ali Khademhosseini, and Malcolm Xing**

An Alkaline Based Method for Generating Crystalline, Strong, and Shape Memory Polyvinyl Alcohol Biomaterials

Mohammad Ali Darabi [1,2,3,4,5], Ph.D.

Ali Khosrozadeh [4, 6], Ph.D.

Ying Wang [7]

Nureddin Ashammakhi [1,2,3] M.D., F.R.C.S.Ed., Ph.D.

Halima Alem [1,2,8], Ph.D.

Ahmet Erdem [1,2,9,10], Ph.D.

Qiang Chang [4]

Kaige Xu [4]

Yuqing Liu [4], Ph.D.

Gaoxing Luo [7], Ph.D.

Ali Khademhosseini [1,2,3,5,11,*], Ph.D.

Malcolm Xing [4,*], Ph.D.

[1]Center for Minimally Invasive Therapeutics (C-MIT), University of California, Los Angeles, California, USA
[2]Department of Bioengineering, University of California, Los Angeles, California, USA
[3]Department of Radiological Sciences, David Geffen School of Medicine, University of California, Los Angeles, California, USA
[4]Department of Mechanical Engineering, University of Manitoba, Winnipeg, R3T 5V6 Canada
[5]Terasaki Institute for Biomedical Innovation, Los Angeles, California, 90024, USA
[6]Department of Physical & Environmental Sciences, University of Toronto Scarborough, Toronto, Ontario, Canada
[7]Institute of Burn Research; State Key Lab of Trauma Burns and Combined Injury, Southwest Hospital, Third Military Medical University, Chongqing, 400038 China
[8]Université de Lorraine, CNRS, Institut Jean Lamour (UMR 7198), Campus Artem 2 allée André Guinier-BP 50840, F54011 Nancy Cedex, France
[9]Department of Chemistry, Kocaeli University, Umuttepe Campus, 41380, Kocaeli, Turkey
[10]Department of Biomedical Engineering, Kocaeli University, Umuttepe Campus, 41380, Kocaeli, Turkey
[11]Department of Chemical Engineering, University of California, Los Angeles, California, USA

(*) Corresponding authors

1. Materials and methods

Materials: All chemicals were used without further purification. PVA (Mw: 205 000 g/mol, 85% hydrolyzed, Aladdin). MWCNTs (Boyu Gaoke, Beijing, China), NaOH (VWR), KOH (American Type Culture Collection), LiOH (Sigma-Aldrich), alginate acid sodium (Alfa Aesar Co.), and fluorescein sodium salt (Sigma-Aldrich).

Characterization of mechanical properties: To investigate the mechanical properties of the PVA membranes, tensile tests were carried out at room temperature with a material testing machine (universal testing machine; WDW-02, SHIWI Instruments Inc., China) with a 200 N load cell.

Characterization of chemical properties: The chemical structure of PVA and PVA-H were analyzed with proton nuclear magnetic resonance (^{1}H NMR) spectroscopy, and Fourier transformed infrared (FTIR). To collect the FTIR spectrum of samples, Thermo Nicolet iS10 FTIR Spectrometer was used. ^{1}H NMR spectra of PVA and PVA-H (dissolved in DMSO-d_6 at 80 °C with a concentration of 10 mg/mL) were collected with a Varian Inova 500 NMR at room temperature.

Characterization of thermal properties: The thermal properties of PVA and PVA-H were investigated using a differential scanning calorimeter (DSC) (DSC 8500; Perkin-Elmer) and a thermogravimetric analyzer (Pyris 1 TGA; Perkin-Elmer). To prepare samples for DSC, the dried samples were weighed (between 2.6–2.9 mg) in standard aluminum pans, sealed with lids, heated at the rate of 20 °C/min from 25 °C to 300 °C using nitrogen as a purge gas. For thermogravimetric analysis (TGA), samples were heated in a ceramic pan at the rate of 20 °C/min from 30 °C to 650 °C under a constant stream of nitrogen at a flow rate of 20 mL/min (the dried samples were weighed between 2.8–3.1 mg).

The PVA and PVA-H crystallinity $\overline{X_c}$ (%) was calculated following the Eq 1:

$$\overline{X_c} = \frac{\Delta H_f\ (T_m)}{\Delta H_f^0 (T_m^0)} \qquad \text{Eq. 1}$$

The $\Delta H_f\ (T_m)$ is the enthalpy fusion of the PVA at the melting point T_m as measured by DSC for the PVA (or PVA-H). $\Delta H_f^0 (T_m^0)$ is the enthalpy fusion of the 100% crystalline PVA [1], $\Delta H_f^0 (T_m^0) = 138.60\, J.g^{-1}$.

X-ray diffraction measurement: Panalytical X'Pert Pro X-ray Powder Diffractometer was used to measure the crystallinity. The machine was run with a generator at 40 mA and 45 kV.

Characterization of electrical properties: The resistance was measured with a two-probes digital multimeter (VICTOR 86E, digital multimeter).

Optical imaging: Optical microscope, SK2009, ShenZhen Saike Digital Technology Development Co., Ltd.

Contact angle measurement: JY-PHA, Dongguan Shengding Precision Instrument Co., Ltd.

3D Printing instrument: A conventional 3D printing was customized, and PVA based solution or alginate was printed with the applied pressure.

Morphology Characterization: The microstructure and morphology of PVA-H were characterized by scanning electron microscopy (SEM, JSM-5900LV, JEOL, Japan) or (JEOL-5900 scanning electron microscope).

2. Experimental conditions

Preparation of PVA solution: PVA was dissolved in distilled at 90 °C under stirring until the solution became homogeneous (100 mg/ml unless otherwise stated).

PVA-Hs films with 0.3 mm thickness: 30 mL of a 100 mg/mL solution of PVA was poured into a large Petri dish, and it was dried at the ambient temperature to form a dried PVA film. After gelation using a high-concentration solution of NaOH, the as-prepared PVA-H was washed with distilled water, cut into strips for tensile tests, and stored in water.

Sample sizes and the tensile test conditions used for Figure 1G. PVA-Hs were crosslinked with different concentrations of NaOH including 1, 3 and 6 M, and cut into strips for tensile experiments with following sizes: for 1 M (15, 5, 0.05 mm); 3, 5 and 6 M (15, 5, 0.1 mm). The test speed was 10 mm/min.

Experimental conditions used for Figure 1L. A medical glove, cut into a circular membrane with a thickness of 0.25 mm, and two PVA-H membranes with 0.08 and 0.2 mm thicknesses were tightened in a customized holder as seen in Figure 1K. The force-displacement graph was recorded with 50 mm/min test speed.

Samples sizes used for Figure 1M-O. Designed PVA-Hs have the capability to be uniformly incorporated different nanomaterials. To prepare composite films, magnetic nanoparticles (MNPs), graphene or CNTs were added to the PVA solution, followed by casting and crosslinking. It is noted that the wt% of elements PVA and nanomaterials can be tuned depending on the required properties. According to the Figure 1M, combining PVA with MNPs led to a stretchable magnetic membrane which can be used as a magnetic actuator. 0.1 g MNPs were mixed with 5 mL of PVA solution, sonicated and stirred, before casting on a petri dish to form a film. Afterward, the dried film was crosslinked using NaOH solution. Video S2 shows how magnetic membrane can dance by a moving magnet. Figure 1N shows graphene was uniformly distributed in the PVA network in a PVA/graphene membrane. Figure 1O displays a nanomembrane made with PVA/CNT solution (PVA with 10 mg/mL of CNT). The solution was poured on a dish, dried overnight, and made a homogenous film following by crosslinking.

Experiment condition for Figure 2B, 2C and Figure S7. A PVA-H membrane with a thickness of 0.3 mm was cut into strips with a width of 10 mm and a length of 13 cm. After immersing the strip in 6M NaOH solution for 5 min, it was used for artificial muscle tests (Figure 2B, 2C and Figure S7) and measuring the contraction force (Figure 2C(3)). After soaking the strip in NaOH solution, it was manually stretched and was attached to a 0.5 kg weight reaching the final length of 38 cm (Figure 2C (1,2)).

Experimental condition for Figure 2C(3). Similarly, after immersing the strip in NaOH solution, it was elongated to L_1 =32 cm and positioned between tensile machine grips. After calibration, the strip was extended manually to reach 5 N preload, to mimic weight hanging. The force was recorded while the water was continuously added to the both sides of the PVA-H strip.

Preparation of PVA composite in Figure 3A-D. First solution: 10 wt% AgNP and 10 wt% PVA blended; second solution: 10 wt% PVA; third solution: 8.8 wt% CNT and 10wt% PVA blended. 0.5 g of each solution were casted on a small petri dish sequentially. First solution was casted and allow it to totally dry at room temperature, then, similarly second and third solutions were poured and air dried. After drying, the rod with coated layers was soaked in NaOH solution for 15 min for crosslinking. The composite membrane was easily peeled off from the metal rod and washed with water.

Swelling ratio. Swelling ratio was calculated according to following formula: Swelling ratio= $[W_f - W_0/W_0]$; in which, W_f is the weight of PVA-H in fully swollen state and W_0 denotes the one for dried state.

Tubes and catheters made in Figure 3. To make small tubes, a metal rod was immersed in PVA solution and rotated in a customized motor with applied drying fan, creating a uniform layer of PVA coating. By using a high rotation speed, we can reach a uniform coating. The process of coating was repeated to reach the desired thickness. Afterwards, the dried PVA tube was crosslinked by 6M NaOH solution followed by washing with water.

Measuring burst pressure of tubes and balloon diameter (Figure 3). Tubes were attached and sealed to a pipe and filled with water and connected to a compressor. With increasing the pressure, tubes were blown and the burst pressure, when they performed excessive expansion and ultimately explosion, were recorded.

Preparation of an injectable electronic (Figure 4A). PVA can serve as stabilizer to effectively distribute CNTs in the solution without coagulation. The amount of CNT can be tuned to obtain required conductivity. First, 100 mg PVA was dissolved in 5 mL water at 90 °C, followed by adding 50 mg CNT. The mixture was further stirred and sonicated for 1 h. Afterwards, 400 mg PVA was dissolved in the mixture under stirring at 90 °C (to reach PVA concentration of 100 mg/mL), and the mixture was further shaken in moving bath at 60 °C overnight. This PVA-CNT ink was used as printing ink to print an electronic mesh on the back of a petri dish as seen in Figure 4A. When prints were dried, they were soaked in NaOH solution followed by immersion in water to physically crosslink PVA and CNT networks.

Preparation of the stretchable electronic in Figure 4B. To make the substrate, 5 ml of 100 mg/mL PVA was poured in a petri dish and left overnight at room temperature to dry. Then, PVA-CNT was printed on the dried PVA substrate and let dry for several minutes, followed by immersion in NaOH solution and water in sequence to crosslink PVA.

Preparation of microfluidic channels Figure 4C and D. 20 ml of PVA solution (100 mg/mL) was poured in a large Petri dish and dried overnight at the ambient temperature. Then, a 5 wt% alginate solution was prepared for printing on the PVA membrane as sacrificial material. After printing the alginate patterns, the dried PVA with 3D printed alginate was covered with another layer of PVA solution, followed by drying at room temperature. Then, the dried membrane was immersed in NaOH solution for 15 min. After the PVA gelation, the membrane was washed with water and the printed alginate was readily removed from edges be pressing the membrane.

3. Literature

Table S1. Comparison of the performance of selected PVA-Hs in the literature with our work.

Ref	Elastic modulus MPa (strain%)	Tensile strength (MPa)	Elongation at break (%)	Swelling ratio (%) in water	Water Content (%)	Method
[2] 2017	___	1.14	550	68	86	mixed-solvent (DMSO-DMF 4:6) heating at 130 °C and cooling
[3] 2011	0.05-1	----	5	80-115	----	freeze-thaw (6 Cycles)
[4] 2017	0.35	2	800	----	84	Graphene oxide/PVA, freeze-thaw (9 Cycles)
[5] 2014	5	2.5	160	60	---	Polyacrylamide/PVA, annealing and rehydrating
[6] 2015	---	3.11	525	390	65	PVA/Melamine, freeze-thaw (3 Cycles)
[7] 2012	0.35	0.28	410	---	----	freeze-thaw (1 cycle)
[8] 2016	---	0.65	270	---	1000	freeze-thaw (3 Cycles)
[9] 2002	0.84	0.024	---	---	----	photo-crosslinking PVA-peptide RGDS
This work	1.7-25	13-16	(50-350)	80-100	33	NaOH treatment

4. *In vitro* and *in vivo* experimental conditions

4.1. Cytocompatibility tests

HaCaT and 3T3 cell lines were cultured with RPMI (Hyclone, USA) and 10% fetal bovine serum (Gibco, USA) in a 5% CO_2 incubator at 37 °C. The HaCaT and 3T3 cells were seeded in 96-well plates at $5x10^3$ /100 µL per well for measuring methabolic activity and in 24-well plates containing cover glasses at $1x10^4$ /300 µL per well for live/dead assay. For toxicity assay, the culture medium was replaced with the medium extracted from PVA-H after 24 h of incubation, and the untreated medium was used as a blank control. The cells in the 96-well plate were assessed using a cell counting kit (CCK-8, Dojindo, Kyushu, Japan) test on days 1, 3 and 5 after seeding. At each time point, the medium was replaced with RPMI, and 10 µL of CCK-8 solution was added to each well, followed by incubation at 37 °C for 2 h. The mean

optical density (O.D) value was determined at 450 nm using an enzyme-linked immunosorbent assay reader (Thermo Varioskan Flash, USA), and the cell viability of cells on cover glasses was tested by cell live/dead kit (Invitrogen, USA). The relevant reagent added to incubation at 37 ℃ for 20 min, and the resultant images were observed under laser confocal (Zeiss LSM780, Germany).

4.2. Anti-fouling test

Protein adhesion. Bovine serum albumin (BSA, life, USA) was used as protein model in this experiment. PVA membranes and cover glasses (as the control group) were washed three times with deionized water and incubated with BSA solution (2 mg/ml) and FITC-BSA (1 mg/ml) solution at 37 ℃ for 6 h. The membranes were rinsed with deionized water for three times, and the adhesion protein was quantified by BCA kit, the adhesion FITC-BSA was observed by fluorescence microscope (600B, Olympus,Japan).

Bacterial adhesion. Expend E.coli [(ATCC 25922)] was cultured at 37 ℃ for 12 h (250 rpm), and then we adjusted the final density of bacteria liquid to 1×10^{6} cfu/ml. PVA membranes and cover glasses (control) were washed for three times with deionized water and put into a 24-well plater with bacteria liquid (500 μL) dropping on the sample surface, and then incubated at 37 ℃ for 4 h. The membranes were rinsed with deionized water for three times, and added 1ml deionized water to ultrasonic 10 minutes, take 100 μl plated and incubated at 37 ℃ for 14 h.

4.3. Hemocompatibility

Citrated fresh human blood (anticoagulation to blood radio, 1:9) and purified counting platelets ($1x10^{12}$ units / ml) were provided by Department of blood transfusion, Southwest Hospital, Chongqing, china. In hemolysis assay, PVA membranes (1 cm x 2.5 cm) were washed for five times with deionized water and then rinsed with normal saline. The samples were incubated at 37 ℃ for 30 min with 10 ml normal saline, and then 200 μl diluted whole blood (whole blood: saline; 8: 10) were added and incubated at 37 ℃ for 1 h. The normal saline was used as negative control and the distilled water was used as positive control. The tubes were centrifuged for 10 min at 1500 rpm and then the optical density of the supernatant fluid was read by Microplate reader (Thermo, varioskan flash, USA) at 545 nm. The HR was calculated according to the following formula:

HR% = [ODs - ODn] / [ODp - ODn] ×100%

ODs, ODn, ODp were the corresponding OD values of sample, negative control and positive control groups. Measurements were repeated three times for each group.

4.4. Subcutaneous embedding *in vivo* and H-E staining

The membranes were punched into 6-mm diameter discs and sterilized. After general anesthesia with 1% pento-barbital, two mice were operated on. The dorsal surface was shaved and cleaned with 75% alcohol. Using surgical knife, three subcutaneous pockets were created through two separate neat openings of 1-cm in length lateral to the spine. One PVA membrane was implanted into each of the first two pockets and anchored to surrounding connective tissue. The wounds were closed using sutures. In the third pocket, no membrane was used to serve as control. The mice were euthanized four weeks postoperatively to remove the skin tissues where the membranes were implanted. Tissue slices were embedded in paraffin, dyed and stained using H&E staining.

5. The effect of using different alkali metal hydroxides on mechanical properties, crystallinity and morphology of PVA-Hs

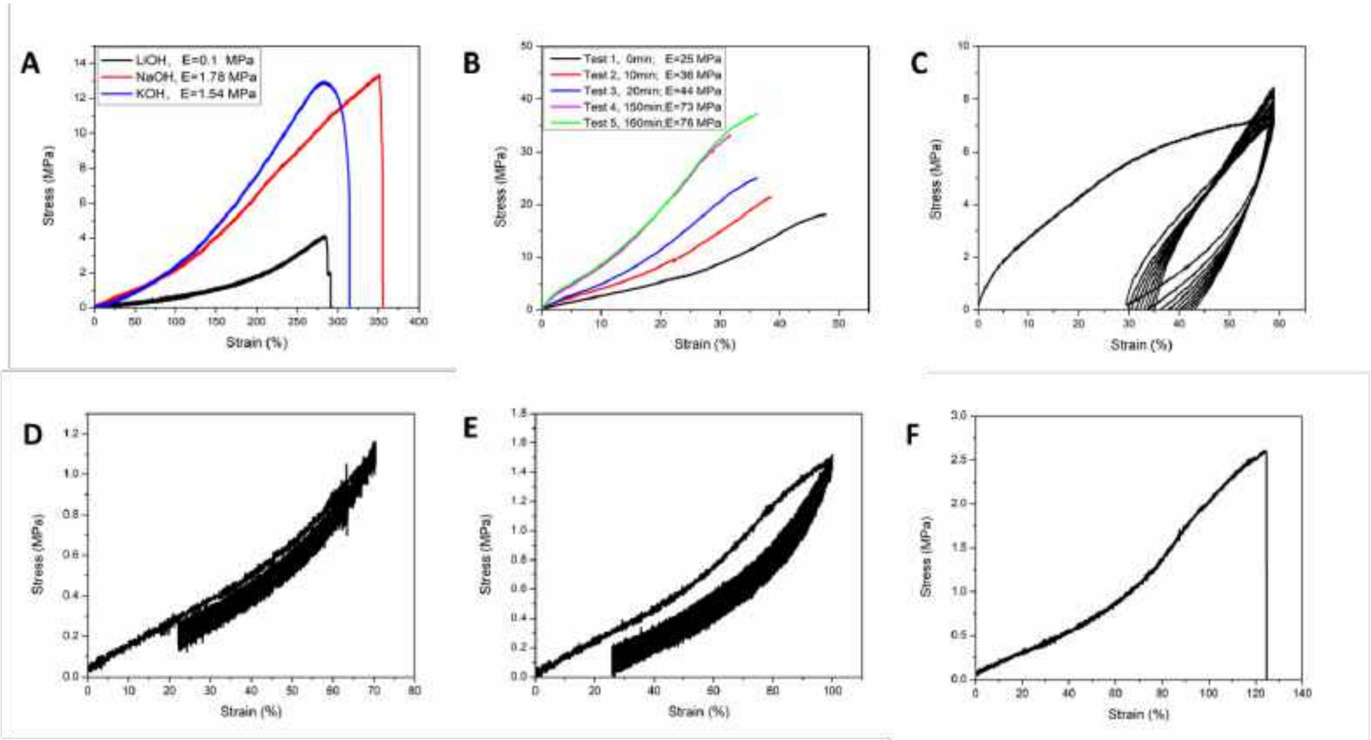

Figure S1. (A) Stress-strain curves and elastic moduli of PVA-Hs made with KOH, NaOH and LiOH (sample sizes for KOH and NaOH were 15, 5, 0.1; and, for LiOH: was 15, 5, 0.05 (length, width and thickness (mm)), and the test speed was 10 mm/min). (B) Tensile test results for a PVA-H crosslinked with NaOH and tested for 5 times in different drying conditions (sample size: 115, 10, 0.3 mm, test speed: 5 mm/min); for example, Test 5 is a PVA-H which was air dried

for 150 min. (C) Cyclic tensile test under room temperature condition (sample size: 17, 5, 0.1 mm, test speed: 20 mm/min. (D, E) Cyclic tensile tests for a PVA-H when a humidifier was used to avoid dehydration during the test (sample size: 25, 5, 0.1 mm, test speed: 20 mm/min); cyclic tensile test was performed in the strain ranges of (D) 22%-70% and (E) 25%-100%. (F) stress-strain curve of the same sample used in D and E under moisturizing condition.

It should be noted that all samples (0.1 mm thickness) in tensile measurements were ruptured at the connection edge due to stress concentration of tightly closed grips, and thus these results show minimal strength and elongation of the PVA-H films. Interestingly, the as-prepared PVA films can be even stronger by increasing their thickness more than 0.1 mm. For example, the elastic modulus increases from 1.8 to 25.0 MPa and the stress at 50% strain increases from 1.1 to 18 MPa when the thickness of PVA film grows from 0.1 mm to 0.3 mm (Figure S1A and B). The potential underlying reason is that the thinner film is homogenous while the thicker one is anisotropic in the thickness direction due to its much denser PVA network deep inside. In other words, the top and bottom of the 0.3 mm film absorb more water compared to inner PVA networks far from surfaces, which results in denser crystalline structure around mid-plane of the film and thus larger overall elastic modulus. This enhancement in the mechanical strength of thicker PVA films is attributed to denser mid-plane PVA networks, i.e. anisotropy without addition of a secondary material.

To investigate the effect of dehydration on the mechanical properties of PVA-H, a PVA-H strip (0.3 mm thickness) was tested for five consequent times with a speed of 5 mm/min immediately after removing from water (Figure S1B). It should be noted that the strip was slipped from the clips without rupture in every test, and thus the strip was immediately installed for the next tensile test. This film with 0.3 mm thickness is so strong that the slippage occurred in all tests (Figure S1B) since the large tensile force exceeded the grip ability to restrain the samples. According to Figure S1B, the fourth and fifth test (the most dried) showed the highest strength (stress before slippage) and elastic modulus, while the first test (the least dried) showed the lowest strength. This observation implies that the mechanical strength of the PVA-H increases with dehydration (it becomes stiffer with loosing water). This is because the lower water content of a PVA-H leads to denser assembly of PVA chains [10]. The fact that the tensile test of the fifth test (160 min out of water) followed the same trend as the fourth test (150 min) indicates that the dehydration further than 150 min (already dried) has negligible effect on the mechanical performance of this strip and that the strip is reusable without performance change.

To investigate the behavior of the PVA-H during cyclic tensile test, another strip (0.1 mm thickness) was stretched-released for 10 cycles from 30% to 58% strain (Figure S1C). During 10 cycles in 8 min, the continuous dehydration of the strip is evident the gradual increase of elastic modulus and mechanical strength. For example, the maximum stress of tenth cycle was 14% more than that of the first cycle as seen in Figure S1C. In figure S1D and E, the same strip was exposed to water vapor generated by a humidifier to prevent dehydration of the strip during the cyclic test. In contrast to the cyclic tensile test carried out in the room condition (cyclic tensile test), the strip maintained its mechanical properties when it was exposed to the humidifier as indicated by the mere difference between first and last cycles. The same sample was further tested in the presence of humidity to show its stress-strain behavior in the fully hydrated state (Figure S1F).

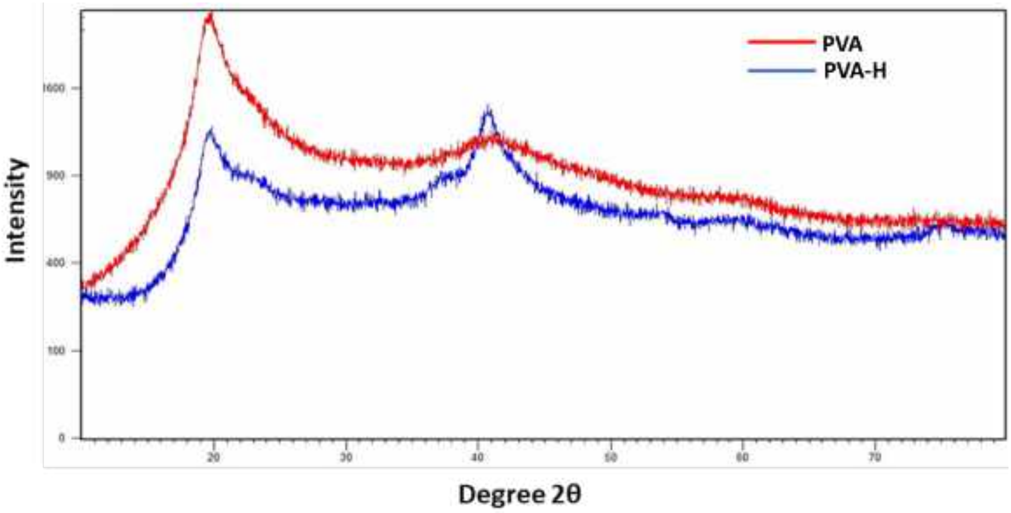

Figure S2. XRD patterns of PVA films before and after crosslinking with NaOH.

Figure S2 presents X-ray diffraction (XRD) patterns of PVA and PVA-H. Two halos centered at 2θ ≈ 20° and 41° (d= 4.45 and 2.17, respectively) are clearly seen for both PVA and PVA-H, indicating characteristics of crystalline PVA. The peak at 2θ ≈ 20° is attributed to the diffraction of the (101) plane [11]. The change in the crystallinity degree is confirmed by the increase of the area of this diffraction peak shown in Figure S2, which is the result of larger crystallites in PVA-H as shown by Hong et al [12].

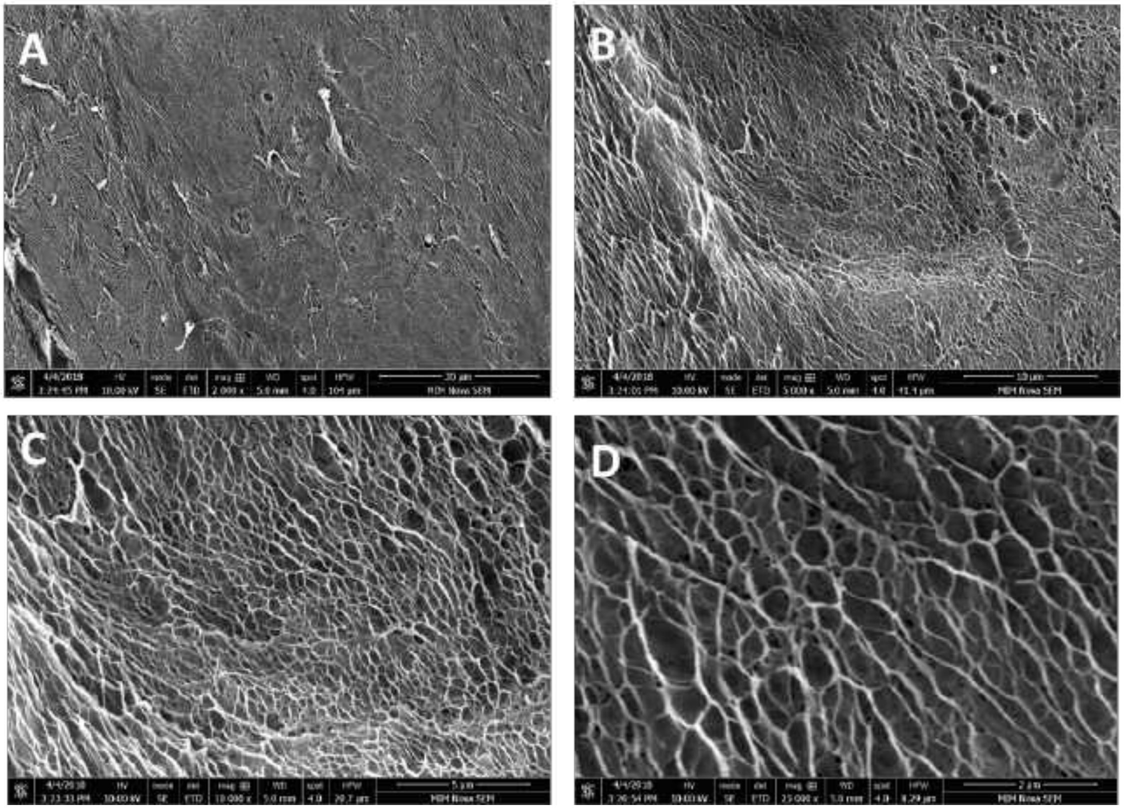

Figure S3. SEM images of a PVA-H film, which was freeze dried and gold coated before SEM.

6. Dissociation of PVA-H with heating in water

To investigate the temperature stability of the PVA-Hs, they were immersed in water with a certain temperature. A PVA-H was stable at temperatures below 65 °C, but it started to change at around 70 °C. To probe the effect of heating, a PVA-H was heated at 70 °C for 30 min, and then it was stored in water at room temperature overnight. It was observed that heating treatment substantially decreased the mechanical properties and increased the swelling ratio from 0.8 to ~9.5. The SEM images in Figure S4 show that the partial dissociation of PVA in water at 70 °C led to macro-sized porosity. Hence, the porosity and the swelling ratio of PVA-Hs can be also tuned by using heat treatment in water (~70 °C). The rate of PVA-H dissociation in water increases with raising the temperature higher than 70 °C, and it will be fully dissolved in water around 90 °C, indicating the recyclability of PVA-based devices.

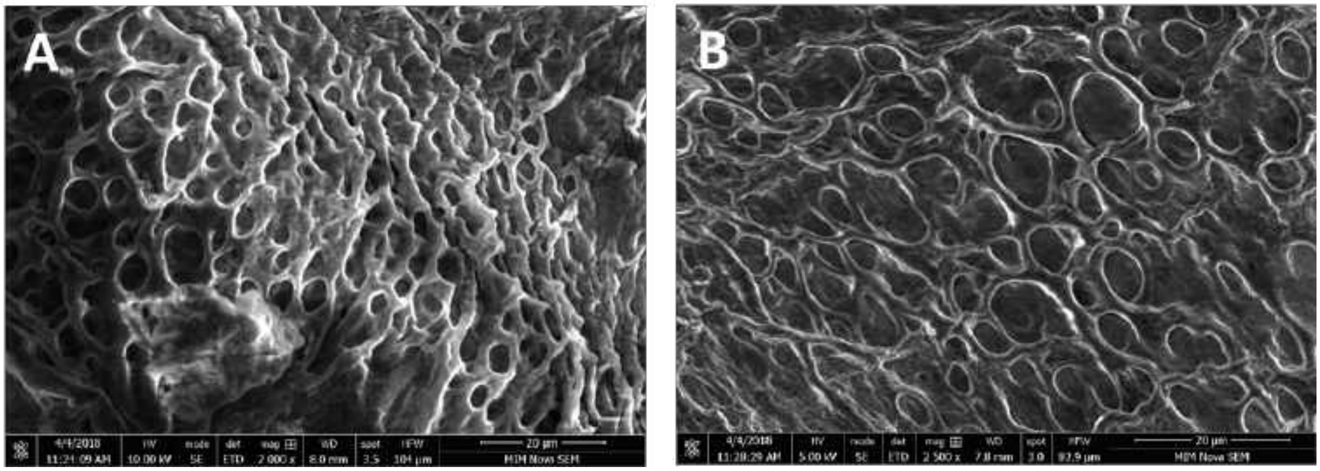

Figure S4. Dissociation of the PVA-H with heating. (A, B) SEM images of a PVA membrane heated at 70 °C for 30 minutes. The size of porous significantly increased for heated samples.

7. PVA-Hs coated with PANI for pH sensing

Polyaniline (PANI) was coated on PVA films using *in situ* polymerization of aniline. Briefly, PVA-H films were soaked in a 70 mL solution of 1 M H_2SO_4 containing 0.32 mL of aniline for 30 min at room temperature. Then, a solution of 266.7 mg of ammonium persulfate (APS) solved in 10 mL of 1 M H_2SO_4 was added to the former solution to initiate the *in situ* polymerization of aniline in the presence of PVA-H films. Following the reaction for 3 h, the PANI-coated PVA-H films were washed with deionized water and stored in water. The mass loading of PANI on the PVA-H films can be increased by repeating the coating process. PANI-coated PVA-H films with one coat (Figure 1P) and two coats of PANI (Figure S5) were prepared. It is observed that PANI can be effectively coated on PVA membranes, offering potential applications in flexible and stretchable energy storage and sensor solutions.

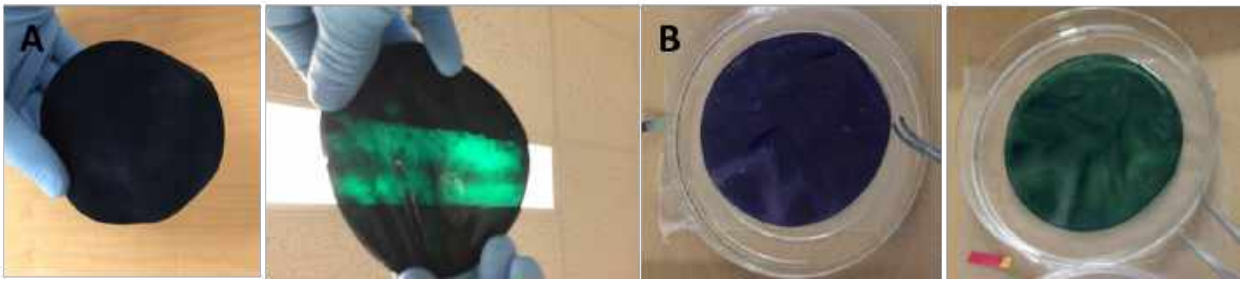

Figure S5. (A) A PVA-H after two coats of PANI. (B) The color of a PANI-coated PVA-H film changes from green to blue/purple when it is transferred between acidic and basic solutions (Video S2).

8. Effect of the molecular weight on PVA-H formation

Our NaOH-induced crosslinking method was used for PVA polymers with different molecular weights including, 1) MW 205,000 (85% hydrolyzed), 2) MW 89,000-98,000 (99% hydrolyzed), 3) MW 31,000-50,000 (98-99% hydrolyzed), 4) MW 13,000-23,000 (98% hydrolyzed) and 5) MW 9,000-10,000 (80% hydrolyzed) (Sigma-Aldrich). We were able to obtain PVA hydrogels for PVA polymers with high molecular weights (i.e. 1, 2, 3), using our method. For the molecular weights less than 31,000, this gelation method was ineffective, i.e. PVA films were degraded in the washing steps. Accordingly, high molecular weight is an important parameter for applying this method. Figure S6A shows the optical images of PVA-Hs formed using PVA polymers with three higher molecular weights. In addition, this experiment reveals that using PVA with higher degree of hydrolysis does not inhibit the crosslinking, since groups #1 and #2 both have high molecular weights, but different hydrolysis ratios and we were able to make strong hydrogels using our approach. Figure S6B also presents the tensile behavior of PVA-Hs made with PVA polymers # 2 and 3. According to the tensile experiment, both molecular weights can result in PVA hydrogels with high mechanical strength more than 3 MPa.

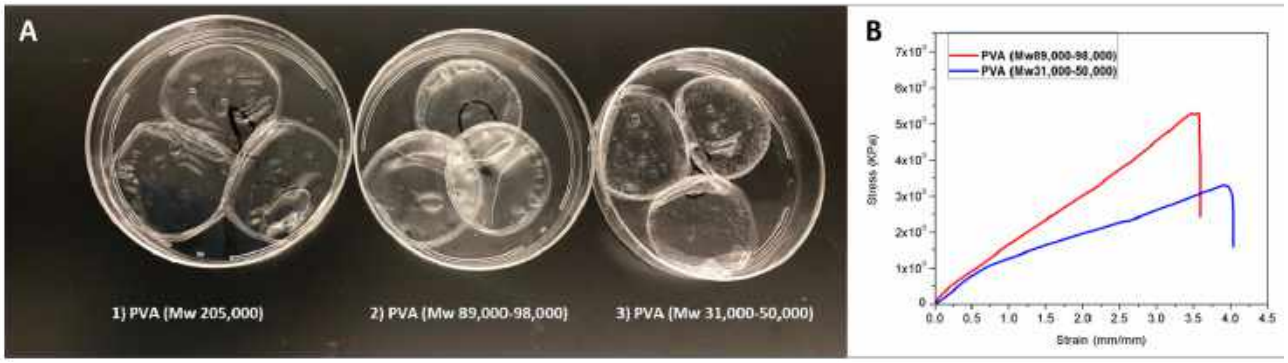

Figure S6. A) Optical photos of PVA-Hs made with different molecular weights using NaOH-induced gelation methods. B) Tensile test results for PVA-Hs with different molecular weights (sample size: 15, 5, 0.2 mm, tests speed: 20 mm/min). Both samples were immersed in water overnight and tested afterwards.

9. Shape memory properties & artificial muscle

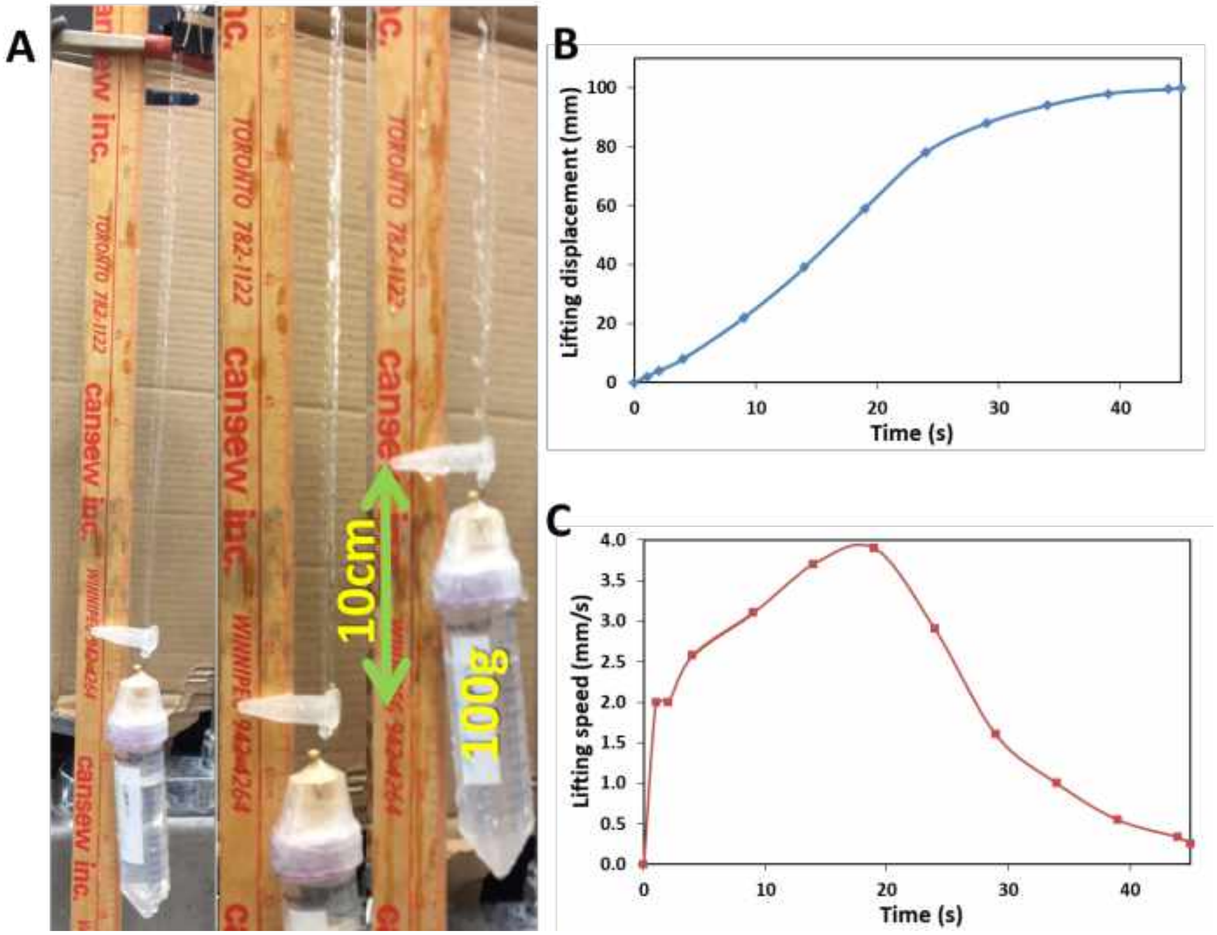

Figure S7. (A) Contraction force due to addition of water can lift 100 g weight up to 10 cm. (B, C) Displacement and velocity versus time during lifting 100 g weight (Video S4).

10. *In vitro* and *in vivo* studies

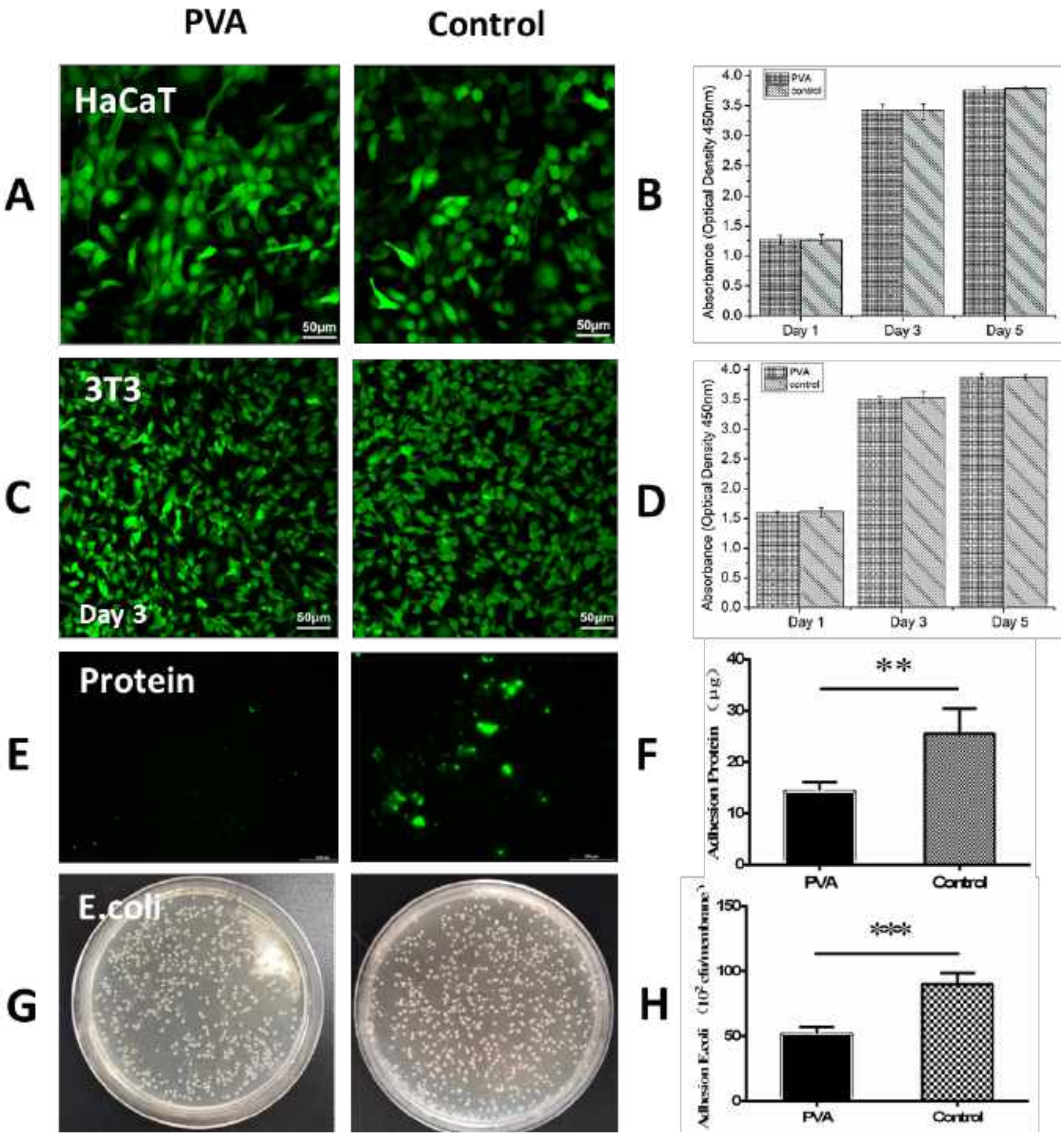

Figure S8. Cytotoxicity assessment of PVA-Hs: (A, C) Confocal microscopy images of HaCaT, 3T3 cells cultured with medium extracting from PVA-Hs at day 3 post seeding. (B, D) The cytotoxicity test and cell viability for HaCaT and 3T3 cells presented by the optical density values were measured by CCK8 assay at days 1, 3 and 5 post seeding. (E) The FITC-BSA protein adhesion on the PVA-H membrane and cover glass (control). (F) The adhesion protein values by BCA kit of each group. (G, H) Anti-fouling surface testing with *E. coli.* (G) Images of detection of survival bacterial CFUs. (H) Quantitative results for the adhered bacterial on membranes (n=3). The differences were considered statistically significant with p<0.05 denoted with an asterisk (*), p<0.01 denoted with an asterisk (**), p<0.001 denoted with an asterisk (***).

Table S2. The hemolysis ratio (HR) of PVA-H membrane

Samples	Optical density at 545 nm	HR%
**Normal saline**	0.0505 ± 0.0007	—
**Distilled water**	0.6010 ± 0.0126	—
**PVA-H**	0.0518 ± 0.0005	0.22

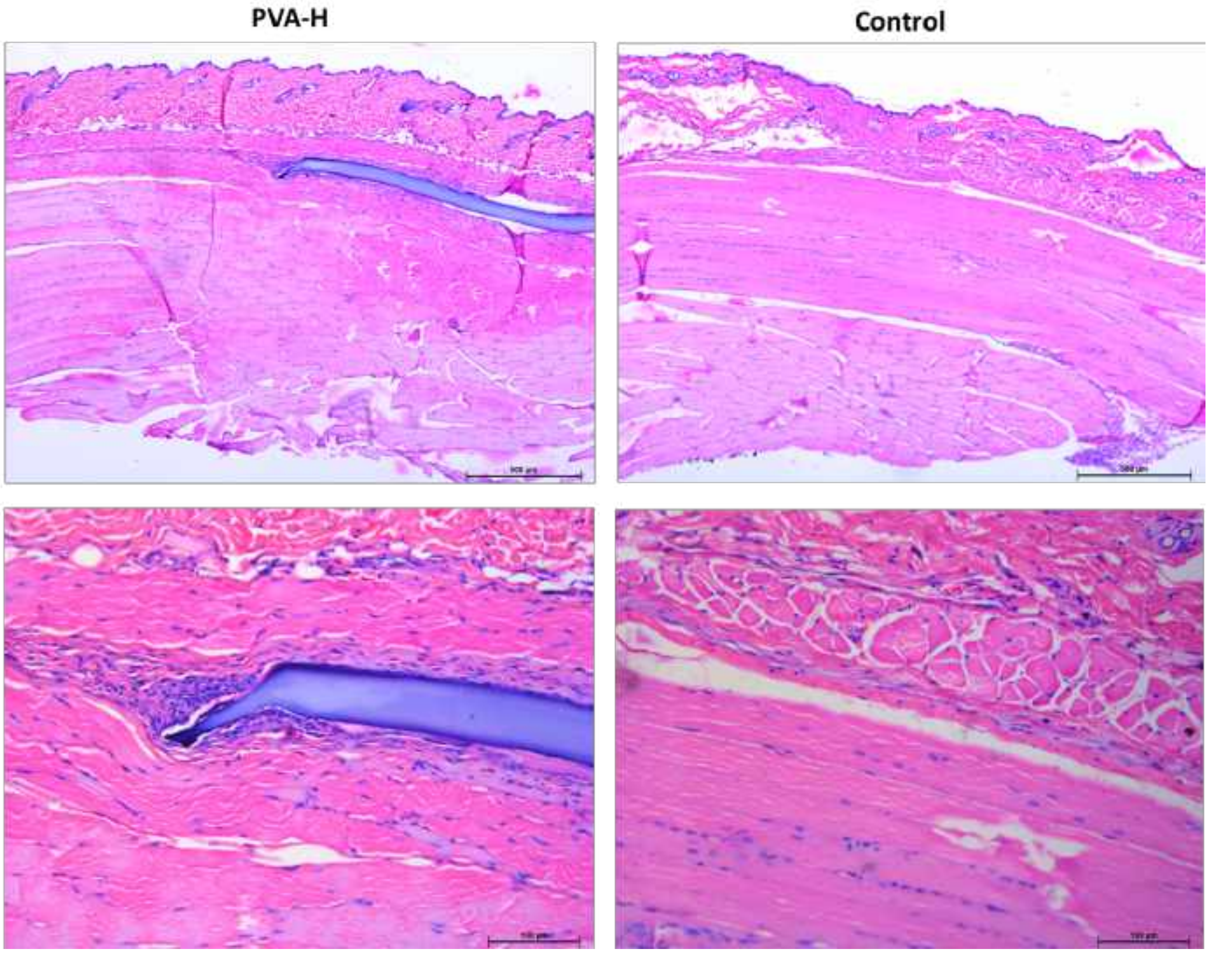

Figure S9. H-E staining of subcutaneous tissue slice of a mouse where the PVA-H membrane was embedded compared to the sham group after four weeks.

11. EDX study

Table 3. Weight percentage of elements obtained from energy dispersive X-ray (EDX) spectroscopy for each layer in the triple-layered membrane (PVA/CNT, PVA and PVA/AgNPs).

Element	PVA Weight %	PVA/CNT Weight %	PVA/AgNPs Weight %
C	52.7	56.0	36.4
O	22.6	14.5	19.5
Au[1]	13.5	16.6	12.7
Pd[1]	11.3	12.9	9.5
Na	0	0	2.8[2]
Ag	0	0	19.0

[1] Elements Au and Pd are the result of gold coating of the sample before conducting SEM.

[2] The small amount of Na is the residual sodium ions after washing.

According to the EDX spectra in Figure 3D, PVA-CNT layer contains highest amount of carbon element compared to other two layers. Ag peak is also appeared in third layer. Accordingly, the presented method enables synthesizing micro- and nano- scale membranes with different materials and properties. Conductivity measured was 30-50 Kohm/cm for PVA-CNT, 400-600 Kohm for AgNPs and 10 Mohm for PVA.

12. Videos

Video S1: Demonstration of resistance of a PVA film against pointed metals.

Video S2: Demonstration of a dried PANI-coated PVA-H ribbon lifting 9 Kg. Demonstration of a PVA/MNPs hybrid hydrogel moving with a magnet. pH detection: the color of a PANI-coated PVA film changes from green to blue/purple when it is transferred between acidic and basic solutions.

Video S3: Demonstration of shape memory effect of PVA-H ribbon: water-assisted elongation recovery of the NaOH-treated and elongated ribbon.

Video S4: Demonstration of artificial muscle behavior of PVA-H ribbon: a weight was lifted by the contraction force generated in the NaOH-treated and elongated ribbon upon supplying water.

Video S5: Demonstration of PVA-H (not/treated with NaOH) performance carrying 9 Kg and recovering the permanent deformation upon adding water.

Video S6: Demonstration of injectable electronics: injection of a printed PVA/CNT hydrogel into water.

Video S7: Demonstration of stretchable electronics: PVA/CNT mesh printed on a PVA-H film.

Video S8: Demonstration of all-PVA microfluidic channels on a PVA-H membrane and tube.

References

[1] N. A. Peppas, E. W. Merrill, *Journal of Applied Polymer Science* **1976**, 20, 1457.

[2] S. Ma, S. Wang, Q. Li, Y. Leng, L. Wang, G.-H. Hu, *Industrial & Engineering Chemistry Research* **2017**, 56, 7971.

[3] J. L. Holloway, K. L. Spiller, A. M. Lowman, G. R. Palmese, *Acta biomaterialia* **2011**, 7, 2477.

[4] J. Chen, X. Shi, L. Ren, Y. Wang, *Carbon* **2017**, 111, 18.

[5] J. Li, Z. Suo, J. J. Vlassak, *Journal of Materials Chemistry B* **2014**, 2, 6708.

[6] G. Li, Q. Yan, H. Xia, Y. Zhao, *ACS Applied Materials & Interfaces* **2015**, 7, 12067.

[7] H. Zhang, H. Xia, Y. Zhao, *ACS Macro Letters* **2012**, 1, 1233.

[8] S. Bhowmick, V. Koul, *Materials science & engineering. C, Materials for biological applications* **2016**, 59, 109.

[9] R. H. Schmedlen, K. S. Masters, J. L. West, *Biomaterials* **2002**, 23, 4325.

[10] H. Kamata, Y. Akagi, Y. Kayasuga-Kariya, U.-i. Chung, T. Sakai, *Science* **2014**, 343, 873.

[11] a) R. Ricciardi, F. Auriemma, C. De Rosa, F. Lauprêtre, *Macromolecules* **2004**, 37, 1921; b) R. Ricciardi, F. Auriemma, C. Gaillet, C. De Rosa, F. Lauprêtre, *Macromolecules* **2004**, 37, 9510.

[12] P. D. Hong, J. H. Chen, H. L. Wu, *Journal of applied polymer science* **1998**, 69, 2477.
